# Challenges in transitioning adolescents and young adults with rheumatologic diseases to adult Care in a Developing Country - the Brazilian experience

**DOI:** 10.1186/s12969-017-0176-y

**Published:** 2017-05-30

**Authors:** Catherine Gusman Anelli, Ana Luiza Mendes Amorim, Fabiane Mitie Osaku, Maria Teresa Terreri, Claudio Arnaldo Len, Andreas Reiff

**Affiliations:** 1 Division of Rheumatology, Department of Pediatrics, Federal University of São Paulo, São Paulo, Brazil; 2 Division of Rheumatology MS 60, Children’s Hospital Los Angeles, Keck School of Medicine, University of Southern California, Los Angeles, USA

**Keywords:** *Transition*, *Brazil*, *Rheumatology*, *Research in health services*

## Abstract

**Background:**

Transition guidelines and recommendations for developing countries are limited and best transition practices in young patients with chronic medical conditions have been poorly examined. This study evaluates transition practices from pediatric to adult rheumatology care in Brazil.

**Methods:**

Practicing pediatric rheumatologists registered in the Brazilian Society of Rheumatology were e-surveyed with SurveyMonkey® using the Chira et al. questionnaire that had been used previously to evaluate transition practices of pediatric rheumatologists from the Childhood Arthritis and Rheumatology Research Alliance (CARRA) in the USA and Canada. The questionnaire was modified to better address specific issues pertaining to the Brazilian health care system.

**Results:**

Seventy-six of 112 (68%) pediatric rheumatologists responded. Only 13% of the respondents reported that they had a well-established transition program and only 14% were satisfied with their current transition process. Eighty percent did not use any specific tools to assess transition readiness. While 43% of respondents considered 18 as the ideal transition age, only a third effectively transitioned their patients at that age while 48% did later. Major hurdles for a successful transition cited by the respondents included emotional attachment to the patients (95%) insufficient training in transition practice (87%), lack of devoted time for transition preparation and process (80%), lack of assistance by pediatric generalists, (77%), and lack of available adult subspecialists (75%). Sixty-seven percent of respondents stated that their program would need more tools/resources to facilitate transition and 59% believed that the development of specific guidelines would be useful to standardize and help with the transition process.

**Conclusions:**

Our study demonstrates that the identified challenges pertaining to transition in Brazilian patients are similar to those reported by pediatric rheumatologists in the United States and Canada. However, the current financial economic pressures affecting Brazil’s health care system may force physicians to deprioritize non emergent care such as transition. A comprehensive understanding of transition issues specific to youth in developing countries and educating not only patients but also health care providers about the importance of a seamless transition process will support the development of transition guidelines and ensure better outcomes of pediatric subspecialty patients.

## Background

As the number and survival rates of children with chronic health care needs and complex functional impairments increase, adult heath care providers are facing new challenges to care for these patients [1–3]. Nowadays 10 years survival rates in chronic pediatric rheumatologic diseases such as juvenile idiopathic arthritis (JIA) and juvenile Systemic Lupus Erythematosus (JSLE) often exceed 95% [4] and a well-structured and effective transition process becomes crucial to guarantee continuity of care and maintain an adequate health status [5–7].

The term transition is defined by the Society of Medicine and Adolescent Health as the “planned and intended change of adolescents and young adults with chronic health care needs from a child centered care to an adult health care system” [2]. In 2007, the American Academy of Pediatrics (AAP) rated "transitioning youth with special health care needs to the adult clinic" among the top 10 priorities of the Academy [8, 9].

Several studies have reported that transition from pediatric to adult health care is often flawed due to limited medical staff training, the lack of a point person responsible for the transition, financial difficulties, as well as anxiety on the part of pediatricians, adolescents and their parents related to the process. Young adults often find it hard to take over the responsibility for their own care and perceive their transition of care as incoherent and dysfunctional [10–13]. In addition, appropriate infrastructure for such specialized health care remains rare and transition practices have not been systematically analyzed [9]. This leads to high dropout rates, loss in follow-up and poor outcomes [6]. Unfortunately, this problem is not unique to pediatric rheumatology but is a concern across all pediatric subspecialties [8, 9].

Recommendations and transition guidelines for developing countries are even more limited and best transition practices in young patients with chronic medical needs have been poorly studied. In a recently published transition study from our university including sixteen HIV-infected adolescents, the authors noted that the patients required more time for the transition process and feelings of disruption, abandonment, or rejection were common. Many expressed the need to meet with their future health care provider prior to transition [14, 15].

About 75% of the Brazilians population relies on the use of the government’s free public national health system that provides free medical services, including hospitalization, surgeries, and drugs prescription [14]. Public hospitals are widely available, and provide free treatment within the four-level health care including emergency services to everyone. Pediatric rheumatology is offered in tertiary and quaternary centers widespread across the country present in greater numbers in capitals and large cities [14].

Since the AAP made transition a focus point, several studies in pediatric subspecialty care including pediatric rheumatology have been conducted and more transition programs have became available [5–7, 16–18]. Studies from the UK found that there was a significant heterogeneity in medical services for adolescents and young adults as well as inappropriate training of the healthcare staff to serve this patient population. [16, 17] In a 25-item survey by the Childhood Arthritis and Rheumatology Research Alliance (CARRA) assessing current transition practices, transition policy awareness, and transitional care barriers, deficiencies in education, resources, and lack of formalized transition processes were noted by all of the respondents [18].

Clemente et al. performed a systematic review on models of transitional care in rheumatic diseases (RMDs), comparing and discussing the methodology and outcomes of different programs that had been implemented and tested in a real life situation. This review was part of a EULAR/PRES initiative to develop recommendations and standards for transitional care for young people with rheumatic diseases and found that the number of structured transitional programs published in the field of RMDs were limited compared to other chronic diseases. The eight transition programs that met the inclusion criteria for best practice analysis were located in North-America and Europe [19].

Data on transition practices in Brazilian pediatric rheumatology does not exist. As a result, transition is often not standardized and occurs abruptly without the proper preparation of the patient, the family and the doctors involved in the patients’ care.

The purpose of this article was to evaluate the transition practices in pediatric rheumatology centers in Brazil using a modified questionnaire that had previously been administered to pediatric rheumatologists in the US and Canada by the Childhood Arthritis and Rheumatology Research Alliance (CARRA).

## Methods

An electronic questionnaire was created through SurveyMonkey® [20], based on a questionnaire previously used by CARRA [18], consisting of 25 questions related to center specific transition practices. We modified this questionnaire by removing 3 questions since they were not applicable to the Brazilian respondents. These 3 questions pertained to “job description” – since all respondents were pediatric rheumatologists, “familiarity with the transition policies of the American Academy of Pediatrics” and “measuring transition outcome”. We also merged 2 questions (question 11 and 12 of the original questionnaire) about “selection of the adult provider” into one and added 2 questions: “How long has the respondent’s rheumatology program existed?” and “Who is responsible for transition?” Certain questions were adapted for Brazilian providers (e.g. “location of practice”, “utilization of transition readiness tools in general instead of asking for specific tools geared towards the US or Canadian population”).

As result, a 23 questionnaire was sent via e-mail to all certified pediatric rheumatologists registered by the Brazilian Society of Rheumatology, which constitutes the majority of all certified PRs in Brazil. To be certified as a pediatric rheumatologist, these professionals had to have at least a year of clinical experience in Pediatric Rheumatology and to successfully pass the Brazilian Society of Rheumatology’s board exam.

Descriptive statistics are presented. When applicable, results from surveyed Brazilian pediatric rheumatologists were compared to responses from the CARRA survey, using comparisons of differences between proportions z test statistical analysis, assuming independence of the 2 cohorts (using a 99% CI and α = 0.01).

## Results

Seventy-six (67.9%) of 112 pediatric rheumatologists registered by the Brazilian Society of Rheumatology answered the questionnaire, representing 43 pediatric rheumatology centers. All of the respondents’ practice settings were located in urban areas and had been well established for 20 years (SD ± 12 years). The average group size consisted of six practicing physicians (SD ± 4.43). Sixty-three percent of the respondents were working in public funded centers linked to a federal or state university system that were part of the public Brazilian Health Care System. Sixty-eight percent of the respondents followed fewer than 10% of patients with private health insurance. For 45% of them transitional youth patients between the ages of 14–21 years made up for a third of the entire patient population.

Table 1 summarizes the main characteristics of the participant’s centers.

**Table 1:** Characteristics of the participant centers

	Answers	N (%)
Affiliation	Federal UC	24 (31.6)
	State UC	24 (31.6)
	Municipal UC	0
	Private UC	13 (17.1)
	None	15 (19.7)
	Unknown	0
	Answers	N (%)
What type of Medical Center are you practicing in?	General (adults and children)	50 (65.8)
	Pediatric (exclusively)	25 (32.9)
	Unknown	1 (1.3)
	Answers	N (%)
How many patients do you follow in your center?	<100 patients	8 (10.5)
	101–500 patients	36 (47.4)
	501–1000 patients	21 (27.6)
	> 1001 patients	6 (7.9)
	Unknown	5 (6.6)
	Answers	N (%)
How many patients do you follow between the age of 14–21 years?	<10%	9 (11.9)
	11–30%	27 (35.5)
	31–50%	23 (30.2)
	> 51%	12 (15.8)
	Unknown	5 (6.6)
	Answers	N (%)
How many of your patients are covered by private health insurance?	<10%	52 (68.4)
	11–30%	11 (14.5)
	31–50%	1 (1.3)
	> 51%	7 (9.2)
	Not informed	5 (6.6)

UC: University center

### Transition practice

Half of the respondents stated that they transitioned 10–50 patients/year to adult care. When asked about the ideal age of starting the transition process, 88% of the respondents agreed that transition preparation should not start before the age of 15 years. Sixty percent considered the ages from 15 to 17 the optimal age to start preparing the patients for transition while 27% considered the ages from 18 to 20 as the ideal age to start preparation. Whereas 43% of respondents considered 18 as the ideal transfer age, only a third reported effectively facilitating the transition at that age. Forty-eight percent stated that transition occurred later, including 18% that transferred patients even after the age of 21 years.

Only 13% of the respondents had a well-established transition program. Transition teams were mainly led by pediatric rheumatologists (38%) and adult rheumatologists (23%) and to a lesser degree by, social workers (19%) and psychologists (17%). However, 61% stated that they had no designated point person.

Transition readiness was assessed by physician’s evaluation of the patient’s knowledge of their illness and current and past treatment. The vast majority (83%) of pediatric rheumatologists stated not to use any specific tools (such as checklists or protocols) to assess transition readiness. The patient’s age, patient request for transition and the need to provide availability for new pediatric patients were the most cited factors that influenced the decision to transition patients. Also, 20% of respondents reported that the fact that their patients had children of their own would influence their decision to transition them earlier. Additionally, 81% answered that the final decision when to transition was solely at the discretion of the pediatric rheumatologist.

The most common practice to facilitate the transition process included providing a comprehensive patient chart summary to the patient and/or adult health care provider at the time of transfer. Due to the lack of a formal transition program, almost 20% of respondents provided a comprehensive chart summary as the only service at the time of transition without any additional formal preparation. Over 60% encouraged the young adult to independently make an appointment with their future adult care provider at least once prior to their final transition. About 75% referred the patients to an adult rheumatologist within the same local medical system.

Tables 2 and 3 summarize the characteristics of the transition team and the main existing transition practices at the participating centers.

**Table 2:** Characteristics of the transition team of participant centers

	Answers	N (%)
	There is no a multidisciplinary team	42 (55.3)
	There is a a multidisciplinary team:	34 (44.7)
Transition Team Composition -at least one of each profession is on the transition team	- Pediatric rheumatologist	29 (85.3)
	- Adult rheumatologist	18 (52.9)
	- Social worker	15 (44.1)
	- Psychologist	13 (38.2)
	- Nurse	5 (14.7)
	- General pediatrician	4 (11.8)
	- Physical therapist	4 (11.8)
	Answers	N (%)
Transition Team Coordination	There is no member assigned to this function.	47 (61.8)
	There is a member assigned to this function:	29 (38.2)
	- Physician	26 (89.6)
	- Psychologist	3 (10.3)

**Table 3:** Transition practices of the respondents

	Answers	N (%)
How many patients do you transition per year?	<10 patients	34 (44.7)
	10–50 patients	41 (54.0)
	>50 patients	1 (1.3)
	Answers	N (%)
What do you consider the ideal age to start the transition process?	<12 years	1 (1.3)
	12–14 years	8 (10.5)
	15–17 years	46 (60.5)
	18–20 years	21 (27.6)
	Answers	N (%)
What do you consider the ideal age to transfer patients?	14 years	4 (5.3%)
	15–17 years	18 (23.7%)
	18 years	33 (43.4%)
	19–20 years	11 (14.5%)
	> 21 years	10 (13.2%)
	Answers	N (%)
Age at which transfer actually happens?	14 years	2 (2.7)
	15–17 years	11 (14.5)
	18 years	26 (34.2)
	19–20 years	23 (30.2)
	> 21 years	14 (18.4)
	Answers	N (%)
Do you have a transition policy?	There is not a formal transition program, but follows an informal protocol to transition patients.	37 (48.7)
	There is not a transition program, but there is interest in implementing one.	27 (35.5)
	There is a formal transition program, well-established and structured.	10 (13.1)
	Transition program under development.	10 (13.1)
	The transition has not been discussed.	3 (4.0)
	There is no need for a transition program at this time.	1 (1.3)
	Answers	N (%)
How do you prepare your patients for transition?	Patient’s knowledge assessment about their own illness, current and past treatments.	50 (65.8)
	Prior visit with an adult rheumatology from the center where the patient will be taken.	48 (63.2)
	Discussion on education, vocation and finding a job.	21 (27.6)
	Patients are assisted by social worker and/or psychologist.	13 (17.1)
	Patient’s knowledge assessment about their health insurance.	11 (14.5)
	Answers	N (%)
How do you facilitate the transition process?	Provide a medical summary of the disease to the patient and/or the center where the patient will be followed.	66 (86.8)
	Schedule the first visit for the patient in the center of adult rheumatology.	30 (39.5)
	Provide copy of the patient’s records.	20 (26.3)
	Development of an individual transition plan.	9 (11.8)
	Provide a map with instructions of how to get to the center of adults where the patient will be followed.	8 (10.5)
	Provide instruction on the health insurance.	7 (9.2)
	Flyers/educational materials.	6 (7.9)
	We do not provide any material.	3 (3.9)
	Other practices.	8 (10.5)
	Answers	N (%)
Do you use any tools for transition?	No tools.	63 (82.9)
	Checklist with objectives to be met by the time of transition.	10 (13.2)
	Protocols that assess the patient’s readiness for transition.	7 (9.2)
	Answers	N (%)
What influences the decision to transfer?	Age of the patient.	73 (96.1)
	Patient request for transition.	28 (36.8)
	Transition patients in order to have place for new patients.	23 (30.3)
	Patient’s family request for transition.	22 (28.9)
	Patient’s disease activity.	20 (26.3)
	Patient having children/starting a family.	15 (19.7)
	Private health insurance status.	8 (10.5)
	Distance between the patient’s residence and its rheumatology center.	7 (9.2)
	Patient getting a job.	5 (6.6)
	Answers	N (%)
Who makes the final decision when to transfer the patient?	The doctor	62 (81.6)
	The patient	4 (5.3)
	The family of the patient	1 (1.3)
	Others	9 (11.8)
	Answers	N (%)
Where do you transfer your patients to?	Adult rheumatology tertiary center linked to the same pediatric rheumatology center.	57 (75.0)
	Secondary specialized medical center not linked to the original center	16 (21,0)
	Private rheumatologist	15 (19.7)
	Patient/family find themselves a specialist	5 (6.6)
	Other	12 (15.8)
	Answers	N (%)
How many times do you allow your patients to return to the pediatric rheumatology center after the transition?	One return.	47 (61.8)
	More than one return.	9 (11.9)
	No returns.	20 (26.3)

### Considerations about the transition

When asked to rank transition priorities, 88% of respondents thought that the pediatric rheumatologist had to be primarily responsible for the transition process. About a half of the participants (47%) considered it very important that the patient had autonomy and were in charge of making their own appointments with the adult health care provider. The most helpful strategies for a successful transition cited by almost all participants included a direct discussion with the patient/family about the transition process during clinic visits (97%), while patient educational groups ranked second (70%), directly educating the patients about the transition over the phone, messaging or via social media (Facebook, Twitter, Instagram) outside regular clinic hours ranked third (52%) and patient questionnaires about the transition process ranked fourth (51%). Interestingly and in contrast to common practice, handing out a printed copy of the medical record and transition specific pamphlets ranked lowest by less than 50% of the respondents (Table 4).

**Table 4:** Transition practices

	Answers	N (%)
Who is primarily responsible for the transition process?	The patient himself.	30 (39.5)
	The family of the patient.	16 (21.1)
	The pediatric rheumatology center.	67 (88.2)
	The adult rheumatology center that will receive the patient.	26 (34.2)
	Answers	N (%)
On a scale from 0 to 5, how important it is that the patient schedule their own appointments?	0	5 (6.6)
	1	3 (4.0)
	2	11 (14.5)
	3	9 (11.8)
	4	12 (15.8)
	5	36 (47.3)
	Answers	N (%)
What resources do you find useful to facilitate transition?	Educational pamphlets targeted to the patient (printed).	Very useful: 36 (47.4)
		A bit useful: 39 (51.3)
		Useless: 1 (1,3)
	Educational pamphlets targeted to the patient (online).	Very useful: 29 (38.1)
		A bit: 43 (56.6)
		Useless: 4 (5.3)
	Educational group sessions regarding the transition process.	Very useful: 53 (69.8)
		A bit useful: 21 (27.6)
		Useless: 2 (2.6)
	Discuss the transition process during medical visits.	Very useful: 72 (97.3)
		A bit useful: 2 (2.7)
		Useless: 0
		No answer: 2
	Questionnaires to assess patients’ knowledge about transition.	Very useful: 39 (51.3)
		A bit useful: 33 (43.4)
		Useless: 4 (5.3)
	Use of direct forms of communication to educate and answer questions: phone calls, emails, text messages, whatsapp, etc.	Very useful: 41 (53.9)
		A bit useful: 31 (40.8)
		Useless: 4 (5.3)
	Use social media to educate and answer questions: Facebook, Twitter, Instagram, others.	Very useful: 38 (50.7)
		A bit useful: 29 (38.7)
		Useless: 8 (10.7)
		No reply: 1
	Printed copy of the medical records.	Very useful: 31 (41.3)
		A bit useful: 40 (53.3)
		Useless: 4 (5.3)
		No reply: 1
	Online version of the medical records.	Very useful: 40 (53.3)
		A bit useful: 27 (36.0)
		Useless: 8 (10.7)
		No reply: 1

### Needs assessment

Only 14% of respondents were satisfied with the way their center currently transitioned patients. Analyzing the factors that respondents considered to be most important in preventing them from successfully transitioning their patients to adult care included the strong emotional attachment that they had formed with their patients over the years (95%), lack of devoted time for transition preparation and process (94%), lack of sufficient knowledge of the transition team how to facilitate the transition process (87%), lack of available adult subspecialists (75%), and lack of assistance by pediatric generalists (77%). In addition, 92% of respondents were concerned about their patients’ inability to take responsibility for their own health care management and their lack of understanding of their diseases (>80%). Sixty-seven percent of participants believed that their center needed more tools/resources to help with transition and 59% believed that the development of specific consensus guidelines would be useful to guide and standardize the process of transition (Table 5).

**Table 5:** Needs Assessment

	Answers	N (%)
What are your needs regarding transition?	Center needs more tools/resources to implement transition.	51 (67.1)
	Development of consensus criteria, with specific guidelines would be useful to guide and standardize the transition process.	45 (59)
	No needs. We are satisfied with the way our center does transition.	11 (14.5%)
	No needs. Our staff is properly trained to deal with transition.	11 (14.5)
	No needs. Our center has sufficient resources and staff to deal with transition.	8 (10.5)
	No needs. Our center devotes sufficient time for transition	5 (6.6%)
	Answers	N (%)
What are the barriers for successful transition?	Lack of general practitioners to assist the transition process.	Does not difficult: 16 (21,0)
		Difficult a bit: 29 (38.2)
		Difficult a lot: 30 (39.5)
		No answer: 1 (1.3)
	Lack of rheumatology centers available for transitioned patients.	Does not difficult: 9 (11.9)
		Difficult a bit: 10 (13.1)
		Difficult a lot: 56 (73.7)
		No answer: 1 (1.3)
	Emotional attachment between the pediatric rheumatology team and the patient/family.	Does not difficult: 4 (5.3)
		Difficult a bit: 32 (42.1)
		Difficult a lot 40 (52.6)
	Lack of patients’ understanding about their disease.	Does not difficult: 13 (17.1)
		Difficult a bit: 36 (47.4)
		Difficult a lot: 27 (35.5)
	The patient’s inability to take responsibility for their own care.	Does not difficult: 5 (6.6)
		Difficult a bit: 36 (47.4)
		Difficult a lot: 34 (44.7)
		No answer: 1 (1.3)
	Lack of devoted time for transition in pediatric rheumatology centers.	Does not difficult: 4 (5.3)
		Difficult a bit: 42 (55.3)
		Difficult a lot: 30 (39.4)
	Lack of knowledge and training of pediatric rheumatology team.	Does not difficult: 9 (11.8)
		Difficult a bit: 37 (48.7)
		Difficult a lot: 29 (38.2)
		No answer: 1 (1.3)

## Discussion

Our data presented here is the first description of transition practices in pediatric rheumatology in Brazil and demonstrates the challenges rheumatologists face in a developing country. These survey results once again confirm the conclusions of the American Academy of Pediatrics that the conditions for transitional youth continue not to be ideal.

We had to adapt the original CARRA questionnaire [18] used by Chira et al. in order to appropriately assess actual transition practices in Brazil as they are experienced by the local providers. We recognize that this change in the questionnaire only allows a limited comparison of the transition data reported in these 2 studies. In addition, studies conducted by surveys in general suffer from incomplete response rates, recall, response and social desirability bias. However, we did not detect any major differences when we compared the responses of Brazilian and US/Canadian pediatric rheumatologists suggesting that these challenges are global and not necessarily linked to the health care system where the transition process occurs. Feedback rates from pediatric rheumatologists were high with a two-thirds response rate comparable to the prior survey conducted by CARRA (67,9% vs 55%, *p* = 0,01778; CI 99%: 0,679, 0,549) suggesting that this data assessment tool may be useful in a developing country. The high rate of participation in our study may be explained by the fact that most respondents worked in a well-established pediatric rheumatology center linked to a university.

Only 13% of the respondents reported to have a structured transition program with few having a multidisciplinary transition team and more frequently without a designated point person compared to the US/Canadian centers (61% vs 30%, *p* = 0; CI 99%: 0,310, 0,618). The most common practice of transition consisted of providing patients and their future adult rheumatologists with a summary of the medical record at the time of patient transfer. US/Canadian and Brazilian pediatric rheumatologists agreed that they should be primarily responsible for the transition process (90% vs 88%, *p* = 0.68916; CI 99%: 0.899, 0.882). Also, the majority of Brazilian pediatric rheumatologists felt that the final decision of when to transfer a patient was determined by them. We found a substantially higher proportion of younger patients transitioning before the age of 21 years in Brazil when compared to the data of CARRA study (82% vs 53%, *p* = 0; CI 99%: 0.816, 0.525). This may be explained by differences between the Brazilian and other health care systems. In addition, 20% of the respondents reported that the fact that their patients had children of their own influenced their decision making to transition these patients earlier, which was substantially less than in the CARRA survey where 55% of the respondents reported that this factor was very important in influencing their decision to transition [18]. Lastly, while 33% of US and Canadian pediatric rheumatologists utilized tools to facilitate the transition process only 17% of the Brazilian rheumatologist made use of these tools, but this difference was not found to be statistically significant (33% vs. 17%, *p* = 0.0114; CI 99%: 0.329, 0.171).

In the absence of well-structured programs and standardized tools, a thorough assessment of the patient’s knowledge of their illness, their current and previous treatments during their clinic visits becomes essential in facilitating a successful transition. Furthermore, in contrast to their US/Canadian colleagues, discussions about vocational resources or strategies how to find a job are not common practice in Brazil (66% vs 27%, *p* = 0; CI 99%: 0.658, 0.263) [18]. In addition, a pre transition visit with the future adult rheumatologist arranged by the patients themselves was felt to be of major importance and further asserting independence and patient autonomy. Lastly there is an increasing awareness that digital media play a crucial role during the transition process and facilitate communication between patient and health care providers. One may speculate that the lack of universal access to such digital media may further impede transition of care in developing countries. However, with growing digitalization of society and increasing access to social media via phone or computers, this may change and become an increasingly important educational and transition tool.

Finally, adult rheumatologists play a crucial role in the adolescent/young adult’s adaptation to the new reality and bring new perspectives to the pediatric visit. Similar to centers in the US and Canada, the lack of adult subspecialists, especially in the Brazilian public health care system where centers of expertise work beyond their capacity, creates a major hurdle to make transition successful. Another major hurdle is the current financial economic pressures affecting Brazil’s health care system that may force physicians to deprioritize non emergent care such as transition. This may explain why more physicians were unaware of transition practices, tools and were lacking the infrastructure to build a transition program.

## Conclusion

In conclusion, transition policies for pediatric rheumatology in Brazil are few and not standardized. This can lead to poor prognosis and unsatisfactory outcomes. We believe that educating not only patients but also adult providers about the importance of a seamless transition process remains a critical issue to serve chronically ill patients population. Our study contributes to a better understanding of transition practices in pediatric rheumatology centers in Brazil, its main weaknesses and failures.

The fact that we did not detect any major differences between the responses of Brazilian and US/Canadian pediatric rheumatologists suggests that concerns related to transition are global. Nevertheless, transition programs should be individualized based on specifics of the local health care systems. The results of our study will hopefully lead to the development of well-structured transition programs and accurate outcomes parameters ensuring a successful transition of pediatric patients to adulthood.

## Abbreviations

CARRA: Childhood Arthritis and Rheumatology Research Alliance
JIA: Juvenile idiopathic arthritis
JSLE: Juvenile Systemic Lupus Erythematosus
